# Associations of apolipoprotein A1, apolipoprotein B, and their ratio with novel cardiometabolic indices in adults with coronary artery disease

**DOI:** 10.3389/fcvm.2026.1893486

**Published:** 2026-09-21

**Authors:** Ping Hu, Nan Lu, Jie Wang, Hongli Dong

**Affiliations:** 1 Image Center, Wuhan Asia Heart Hospital, Wuhan, Hubei, China; 2 Department of Psycho-Cardiology, Beijing Anzhen Hospital Affiliated to Capital Medical University, Beijing, China; 3 Scientific Education Section and Department of Child Healthcare, Affiliated Maternity & Child Health Care Hospital of Nantong University, Nantong, Jiangsu, China

**Keywords:** apolipoprotein A1, apolipoprotein B, atherogenic index, atherogenic index of plasma, cross-sectional study, triglyceride glucose body mass index

## Abstract

**Background:**

Circulating apolipoprotein A1 (ApoA1) and apolipoprotein B (ApoB) have been proposed as vital biomarkers in the pathological process of coronary heart disease (CAD). However, their associations with novel cardiometabolic indices [triglyceride glucose body mass index (TyG-BMI), atherogenic index of plasma (AIP), and atherogenic index (AI)] remain scarce in CAD patients.

**Methods:**

A total of 11,934 patients with CAD (average age: 61.7 years) were included in this hospital-based cross-sectional study. TyG-BMI, AIP, and AI were calculated using the following formulas: TyG index = Ln[FBG (mg/dL) × triglyceride (mg/dL)/2], Log[triglyceride (mg/dL)/high-density lipoprotein cholesterol (HDL-C, mg/dL)], and non-HDL-C (mg/dL)/HDL-C (mg/dL).

**Results:**

Overall, serum ApoA1 was inversely associated with TyG-BMI, AIP, and AI, while ApoB and the ApoB/A1 ratio exhibited positive associations with TyG-BMI, AIP, and AI. In multivariable analysis, ApoA1 was inversely associated with cardiometabolic indices [TyG-BMI_Q(quartile)4 vs. Q1_: 225.800 vs. 228.217, AIP_Q4 vs. Q1_: 0.356 vs. 0.478, AI_Q4 vs. Q1_: 1.928 vs. 2.525]. In contrast, ApoB and the ApoB/A1 ratio were positively associated with cardiometabolic indices. The corresponding values for TyG-BMI, AIP, and AI were 236.772 (vs. 217.018) and 236.724 (vs. 216.873), 0.478 (vs. 0.304) and 0.512 (vs. 0.285), and 3.097 (vs. 1.652) and 3.159 (vs. 1.544) in Q4 group of ApoB and ApoB/A1 ratio compared with Q1 group. Similar results were observed in the subgroup analysis stratified by sex.

**Conclusions:**

ApoA1 was inversely associated with novel cardiometabolic indices (TyG-BMI, AIP, and AI) in CAD patients, while ApoB and the ApoB/A1 ratio demonstrated significant positive associations with these cardiometabolic indices.

## 1 Introduction

Coronary artery disease (CAD) remains the leading global cause of mortality (1). In China, the mortality of CAD reached 135.08 and 148.19/100,000 for urban and rural areas, respectively, in 2021 (2). Hyperlipidemia has been considered the predominant risk factor for the severity of coronary atherosclerosis (3–5). Therapeutic approaches for CAD have expanded from hyperlipidemia to lipid disorders (6). Recent studies have highlighted apolipoprotein A1 (ApoA1) and apolipoprotein B (ApoB) as vital biomarkers in CAD mortality risk (7, 8). However, the associations of ApoA1 and ApoB with novel cardiometabolic indices [*e.g.*, triglyceride glucose body mass index (TyG-BMI), atherogenic index of plasma (AIP), and atherogenic index (AI)] remain scarce in individuals with CAD.

ApoA1, the main component of high-density lipoprotein (HDL), plays a key role in the clearance of excess cholesterol (9). It exerts anti-atherosclerosis effects by modulating oxidative stress and inflammation via interactions with different receptors and transporters (*e.g.*, lecithin cholesterol acyltransferase, scavenger receptor class B type 1, and adenosine triphosphate-binding cassette transporter A1) (9, 10). Moreover, ApoB comprehensively reflects conventional lipid marker information (11) and is considered a more accurate indicator of cardiovascular risk than either total cholesterol or low-density lipoprotein cholesterol (LDL-C) (12). These studies indicate that ApoA1 and ApoB are the important biomarkers in the management of CAD. Meanwhile, earlier studies reported that TyG-BMI, AIP, or AI may serve as novel biomarkers for CAD severity (13–16). TyG-BMI demonstrates superior ability to identify insulin resistance compared with other non-insulin-derived indices (17); furthermore, it exhibits promising utility in the prevention, diagnosis, and management of cardiovascular diseases (18). AIP, correlated with LDL-C particle size, can reflect small dense LDL levels (19, 20) and serves as a robust predictor of CAD, characterizing the balance between anti-atherogenic and pro-atherogenic lipoproteins (21). Moreover, Xia et al. reported that elevated AI was able to better predict the risk of cardiovascular disease (CVD) than HDL-cholesterol (HDL-C) or triacylglycerol (TG) alone (22). However, data examining the associations between ApoA1 and ApoB and novel cardiometabolic indices remain scarce. Bora et al. observed that ApoA1 was an important modulator of adverse AIP values (23). Kaneva et al. clarified that the ApoB/A1 ratio, a biomarker of cardiovascular risk, was correlated with AIP (24). Previous studies also indicated that there may be a racial association between apolipoproteins and serum lipid parameters (25–27). Nonetheless, no study has yet assessed the associations of ApoA1 and ApoB with TyG-BMI, AIP, and AI in patients with CAD. Moreover, noteworthy differences have been found in the levels of blood lipids among the CAD and non-CAD populations (28). Therefore, it remains unclear whether ApoA1 and ApoB are associated with TyG-BMI, AIP, and AI in patients with CAD.

In this study, we aimed to identify the associations of ApoA1, B, and their ratio with novel cardiometabolic indices (e.g., TyG-BMI, AIP, and AI) in adults with CAD.

## 2 Materials and methods

### 2.1 Subjects

The medical records of 11,934 patients with CAD (average age: 61.7 years) were analyzed using a cross-sectional study design. All subjects were recruited from Wuhan Asia Heart Hospital between November 2016 and December 2019. CAD was diagnosed if one of the following criteria was met: (a) history of coronary intervention, angina pectoris, or myocardial infarction; (b) coronary angiography showed vascular stenosis >50%; and (c) electrocardiogram displayed obvious myocardial infarction (29). The present study was performed in accordance with the 1964 Declaration of Helsinki and its later amendments. Approval to undertake this study was granted by the Ethics Committee of Wuhan Asia Heart Hospital (2016–B008). Informed consent was obtained from all subjects.

### 2.2 Epidemiological data and laboratory measurements

Detailed sociodemographic information was collected, such as sex, age, smoking status, alcohol consumption, height, weight, and use of antihypotensive or hypoglycemic agents. The BMI of all subjects was calculated as follows: BMI = weight (kg)/height (m^2^). Serum HDL-C and TG were measured using commercial reagents (Beckman Coulter Inc., Brea, CA, USA) and the Denka Seiken enzymatic colorimetric test (Tokyo, Japan), respectively. Blood samples were collected and tested after approximately 8 h of fasting. Fasting blood glucose (FBG) level was determined using a commercial kit (Roche Diagnostics GmbH, China). Serum ApoA1 and ApoB concentrations were detected with a lipoprotein immune kit on an automatic biochemical analyzer. The ApoB/A1 ratio was calculated. Cardiometabolic indices were calculated as follows: TyG index = Ln[FBG (mg/dL) × triglyceride (mg/dL)/2]; TyG-BMI = BMI × TyG index (17, 30); AIP = Log(TG/HDL-C) (31); and AI = non-HDL-C/HDL-C (13).

### 2.3 Statistical analysis

Normally and non-normally distributed continuous data were presented as the mean [± standard deviation (SD)] and the median (25–75th percentile ranges) and analyzed using the t-test and the Kruskal–Wallis test, respectively. Discrete variables were expressed as counts (%) and were analyzed using the chi-square test.

Partial correlation analyses with adjustment for age and sex were conducted to examine the relationships of serum ApoA1, ApoB, and the ApoB/A1 ratio with cardiometabolic indices (e.g., TyG-BMI, AIP, and AI). All subjects were divided into gender-specific quartile groups according to the levels of ApoA1, ApoB, and ApoB/A1 ratio. The higher the quartile levels represented, the higher the corresponding exposure levels. Quartile 1 (Q1), the lowest concentration group, was considered the reference, and Q4 was the highest concentration group. The mean differences between apolipoproteins and cardiometabolic indices were assessed using the analyses of covariance (ANCOVA). Trends among the quartiles of serum ApoA1, ApoB, and the ApoB/A1 ratio were further tested by ANCOVA. The Bonferroni test was performed to evaluate the pairwise comparisons among quartiles. TyG-BMI, AIP, and AI were reported as the mean [± standard error (SE)]. Two models were estimated: Model 1 was adjusted for sex and age. Model 2 was further adjusted for smoking status, alcohol consumption, and use of antihypotensive, lipid-lowering, and hypoglycemic agents. A two-sided *P*-value <0.05 was considered statistically significant. All statistical analyses were performed using the SPSS software program for Windows (IBM SPSS Statistics 21.0, Inc., Armonk, NY, USA).

## 3 Results

### 3.1 General characteristics of all subjects

Baseline clinical and demographic characteristics of total populations—stratified by sex—are presented in Table 1. The average age of men and women was 60.7 ± 10.38 years and 63.4 ± 9.03 years, respectively. Men exhibited higher proportions of alcohol drinking (14.0% vs. 0.7%); smoking (21.7% vs. 1.4%); and higher ApoB/A1 ratios (0.71 vs. 0.65), AIP levels (0.43 vs. 0.38), and AI levels (1.71 vs. 1.50) compared with women. In addition, men had lower proportions of use of antihypotensive (46.9% vs. 50.5%) and hypoglycemic (16.6% vs. 19.6%) agent, and lower ApoA1 (1.13 vs. 1.27 g/L) and ApoB (0.78 vs. 0.80 g/L) concentrations than women (all *P*-values < 0.001).

**Table 1:** The characteristics of subjects with coronary artery disease^a^.

Variables	Total (*n* = 11,934)	Men (*n* = 7,477)	Women (*n* = 4,457)	*P* ^b^
Age, years	61.7 ± 9.98	60.7 ± 10.38	63.4 ± 9.03	**<0.001**
BMI (kg/m^2^)	25.01 ± 4.05	25.11 ± 4.01	24.84 ± 4.11	0.868
Alcohol drinker, *n* (%)	1,077 (9.0)	1,047 (14.0)	30 (0.7)	**<0.001**
Smoker, *n* (%)	1,685 (14.1)	1,621 (21.7)	64 (1.4)	**<0.001**
Antihypotensive agent user, *n* (%)	5,758 (48.2)	3,507 (46.9)	2,251 (50.5)	**<0.001**
Hypoglycemic agent user, *n* (%)	2,114 (17.7)	1,241 (16.6)	873 (19.6)	**<0.001**
Lipid-lowering agent user, *n* (%)	329 (2.8)	129 (2.9)	200 (2.7)	**0.479**
ApoA1 (g/L)	1.18 ± 0.21	1.13 ± 0.19	1.27 ± 0.21	**<0.001**
ApoB (g/L)	0.79 ± 0.23	0.78 ± 0.23	0.80 ± 0.22	**<0.001**
ApoB/A1 ratio	0.69 ± 0.23	0.71 ± 0.24	0.65 ± 0.21	**<0.001**
TyG-BMI	227.61 ± 38.48	228.12 ± 38.21	226.93 ± 38.94	0.157
AIP	0.41 ± 0.30	0.43 ± 0.29	0.38 ± 0.30	**<0.001**
AI^c^	1.64 (0.30–2.98)	1.71 (0.35–3.06)	1.50 (0.22–2.85)	**<0.001**

ApoA1, apolipoprotein A1; ApoB, apolipoprotein B; AI, atherogenic index; AIP, atherogenic index of plasma; TyG-BMI, triglyceride glucose body mass index.

a Values are means ± standard deviations or *n* (%).

b *P*-values are calculated by Student's t-test for the continuous variables and chi-square test for categorical variables.

c Values are median (interquartile range), *P*-values are calculated using Mann–Whitney U test.

Bold values: statistically significant differences.

### 3.2 Partial correlation analysis

After adjusting for sex and age, ApoA1 levels were inversely correlated with TyG-BMI, AIP, and AI (*r*′ range: −0.157 to −0.039; all *P-*values < 0.001). In contrast, ApoB levels and the ApoB/A1 ratio were positively correlated with TyG-BMI, AIP, and AI (*r*′ range: 0.172–0.353; all *P-*values < 0.001) (Table 2).

**Table 2:** Relationships between ApoA1, ApoB, and ApoB/A1 ratio and cardiometabolic indices^a^.

Variables	ApoA1		ApoB		ApoB/A1 ratio	
	*r*′	*P*	*r*′	*P*	*r*′	*P*
TyG-BMI	−0.039	<0.001	0.189	<0.001	0.172	<0.001
AIP	−0.157	<0.001	0.223	<0.001	0.280	<0.001
AI	−0.133	<0.001	0.318	<0.001	0.353	<0.001

ApoA1, apolipoprotein A1; ApoB, apolipoprotein B; AI, atherogenic index; AIP, atherogenic index of plasma; TyG-BMI, triglyceride glucose body mass index.

a Partial correlation analysis, adjusted for age and sex.

### 3.3 Associations of serum ApoA1, ApoB levels, and the ApoB/A1 ratio with cardiometabolic indices

Overall, serum ApoA1 was inversely associated with cardiometabolic indices (TyG-BMI, AIP, and AI; *P*-trends: 0.028 to <0.001), while serum ApoB levels and the ApoB/A1 ratio exhibited the positive associations with TyG-BMI, AIP, and AI (Tables 3–5). In Model 1, after adjustment for sex and age, higher ApoA1 concentrations were associated with lower TyG-BMI, AIP, and AI in the total population. In contrast, ApoB levels and the ApoB/A1 ratio exhibited positive associations with TyG-BMI, AIP, and AI. After further adjustment for the potential covariates in Model 2, serum ApoA1 was inversely associated with cardiometabolic indices [TyG-BMI_Q (quartile) 4 vs. Q1_: 225.800 vs. 228.217, AIP_Q4 vs. Q1_: 0.356 vs. 0.478, AI_Q4 vs. Q1_: 1.928 vs. 2.525]. In contrast, serum ApoB levels and the ApoB/A1 ratio remained positively associated with TyG-BMI, AIP, and AI. The corresponding values for TyG-BMI, AIP, and AI were 236.772 (vs. 217.018) and 236.724 (vs. 216.873), 0.478 (vs. 0.304) and 0.512 (vs. 0.285), and 3.097 (vs. 1.652) and 3.159 (vs. 1.544) in the fourth quartile of ApoB level and the ApoB/A1 ratio compared with the first quartile, respectively (Model 2).

**Table 3:** The associations of ApoA1 and ApoB with TyG-BMI in all participants (mean ± SE).

Variables	Quartiles by ApoA1, ApoB, and their ratio				*P*-trend
	Q1	Q2	Q3	Q4	
ApoA1	0.95 ± 0.11	1.11 ± 0.07	1.23 ± 0.08	1.44 ± 0.16	
n	2,710	2,604	2,804	2,662	
Model 1	229.170 ± 0.73	228.591 ± 0.75	228.010 ± 0.72	**224.651** **±** **0.74**^a^^,^^b^^,^^c^	**<0.001**
Model 2	228.217 ± 0.72	228.123 ± 0.73	228.276 ± 0.70	225.800 ± 0.72	**0.028**
ApoB	0.53 ± 0.08	0.70 ± 0.04	0.84 ± 0.05	1.08 ± 0.16	
n	2,678	2,689	2,700	2,713	
Model 1	217.557 ± 0.73	**226.300** **±** **0.73**^a^	**230.191** **±** **0.73**^a^^,^^b^	**236.274** **±** **0.72**^a^^,^^b^^,^^d^	**<0.001**
Model 2	217.018 ± 0.71	**226.020** **±** **0.70**^a^	**230.503** **±** **0.70**^a^^,^^b^	**236.772** **±** **0.70**^a^^,^^b^^,^^c^	**<0.001**
ApoB/ApoA1 ratio	0.44 ± 0.07	0.59 ± 0.04	0.73 ± 0.06	0.98 ± 0.21	
n	2,704	2,684	2,698	2,694	
Model 1	216.554 ± 0.72	**225.900** **±** **0.72**^a^	**231.329** **±** **0.72**^a^^,^^b^	**236.696** **±** **0.73**^a^^,^^b^^,^^d^	**<0.001**
Model 2	216.873 ± 0.70	**225.594** **±** **0.70**^a^	**231.287** **±** **0.70**^a^^,^^b^	**236.724** **±** **0.71**^a^^,^^b^^,^^d^	**<0.001**

ApoA1, apolipoprotein A1; ApoB, apolipoprotein B; AI, atherogenic index; AIP, atherogenic index of plasma; TyG-BMI, triglyceride glucose body mass index; Q, quartile; SE, standard error. Model 1 was adjusted for sex and age; Model 2 was further adjusted for smoking status, alcohol consumption, and antihypotensive, lipid-lowering, and hypoglycemic agent use.

a *P* < 0.001 compared with Q1.

b *P* < 0.001 compared with Q2.

c *P* < 0.05 compared with Q3.

d *P* < 0.001 compared with Q3.

Bold values: statistically significant differences.

**Table 4:** The associations of ApoA1 and ApoB with AIP in all participants (mean ± SE).

Variables	Quartiles by ApoA1, ApoB, and their ratio				*P*-trend
	Q1	Q2	Q3	Q4	
ApoA1	0.95 ± 0.12	1.11 ± 0.07	1.23 ± 0.08	1.44 ± 0.16	
n	3,062	2,886	3,033	2,953	
Model 1	0.481 ± 0.01	**0.417** **±** **0.01**^a^	**0.391** **±** **0.01**^a^^,^^b^	**0.353** **±** **0.01**^a^^,^^c^^,^^d^	**<0.001**
Model 2	0.478 ± 0.01	**0.417** **±** **0.01**^a^	**0.392** **±** **0.01**^a^^,^^b^	**0.356** **±** **0.01**^a^^,^^c^^,^^d^	**<0.001**
ApoB	0.53 ± 0.08	0.70 ± 0.04	0.84 ± 0.05	1.09 ± 0.16	
n	3,046	2,840	3,057	2,991	
Model 1	0.306 ± 0.01	**0.416** **±** **0.01**^a^	**0.448** **±** **0.01**^a^^,^^c^	**0.476** **±** **0.01**^a^^,^^c^^,^^d^	**<0.001**
Model 2	0.304 ± 0.01	**0.415** **±** **0.01**^a^	**0.449** **±** **0.01**^a^^,^^c^	**0.478** **±** **0.01**^a^^,^^c^^,^^d^	**<0.001**
ApoB/ApoA1 ratio	0.44 ± 0.07	0.59 ± 0.04	0.73 ± 0.06	0.99 ± 0.21	
n	2,982	2,986	2,983	2,983	
Model 1	0.284 ± 0.01	**0.397** **±** **0.01**^a^	**0.453** **±** **0.01**^a^^,^^c^	**0.511** **±** **0.01**^a^^,^^c^^,^^d^	**<0.001**
Model 2	0.285 ± 0.01	**0.395** **±** **0.01**^a^	**0.453** **±** **0.01**^a^^,^^c^	**0.512** **±** **0.01**^a^^,^^c^^,^^d^	**<0.001**

ApoA1, apolipoprotein A1; ApoB, apolipoprotein B; AI, atherogenic index; AIP, atherogenic index of plasma; TyG-BMI, triglyceride glucose body mass index; Q, quartile; SE, standard error. Model 1 was adjusted for sex and age; Model 2 was further adjusted for smoking status, alcohol consumption, and antihypotensive, lipid-lowering, and hypoglycemic agent use.

a *P* < 0.001 compared with Q1.

b *P* < 0.05 compared with Q2.

c *P* < 0.05 compared with Q2.

d *P* < 0.001 compared with Q3.

Bold values: statistically significant differences.

**Table 5:** The associations of ApoA1 and ApoB with AI in all participants (mean ± SE).

Variables	Quartiles by ApoA1, ApoB, and their ratio				*P*-trend
	Q1	Q2	Q3	Q4	
ApoA1	0.93 ± 0.11	1.10 ± 0.07	1.21 ± 0.08	1.40 ± 0.14	
n	2,553	2,556	2,485	2,466	
Model 1	2.524 ± 0.04	**2.382** **±** **0.04**^a^	**2.126** **±** **0.04**^b^^,^^c^	**1.929** **±** **0.04**^b^^,^^c^^,^^d^	**<0.001**
Model 2	2.525 ± 0.04	**2.379** **±** **0.04**^a^	**2.129** **±** **0.04**^b^^,^^c^	**1.928** **±** **0.04**^b^^,^^c^^,^^d^	**<0.001**
ApoB	0.54 ± 0.08	0.72 ± 0.04	0.86 ± 0.05	1.11 ± 0.16	
n	2,552	2,493	2,526	2,489	
Model 1	1.648 ± 0.03	**1.909** **±** **0.03**^b^	**2.328** **±** **0.03**^b^^,^^c^	**3.103** **±** **0.03**^b^^,^^c^^,^^d^	**<0.001**
Model 2	1.652 ± 0.04	**1.911** **±** **0.04**^b^	**2.329** **±** **0.04**^b^^,^^c^	**3.097** **±** **0.04**^b^^,^^c^^,^^d^	**<0.001**
ApoB/ApoA1 ratio	0.46 ± 0.07	0.62 ± 0.04	0.76 ± 0.05	1.01 ± 0.21	
n	2,513	2,516	2,516	2,515	
Model 1	1.543 ± 0.03	1.946 ± 0.03	2.321 ± 0.03	3.165 ± 0.03	**<0.001**
Model 2	1.544 ± 0.03	**1.951** **±** **0.03**^b^	**2.319** **±** **0.03**^b^^,^^c^	**3.159** **±** **0.03**^b^^,^^c^^,^^d^	**<0.001**

ApoA1, apolipoprotein A1; ApoB, apolipoprotein B; AI, atherogenic index; AIP, atherogenic index of plasma; TyG-BMI, triglyceride glucose body mass index; Q, quartile; SE, standard error. Model 1 was adjusted for sex and age; Model 2 was further adjusted for smoking status, alcohol consumption, and antihypotensive, lipid-lowering, and hypoglycemic agent use.

a *P* < 0.05 compared with Q1.

b *P* < 0.001 compared with Q1.

c *P* < 0.05 compared with Q2.

d *P* < 0.001 compared with Q3.

Bold values: statistically significant differences.

### 3.4 Subgroup analysis

To further explore the associations between serum ApoA1, ApoB levels, and cardiometabolic indices, we performed subgroup analyses stratified by sex and obesity. As shown in Supplementary Table S1, elevated ApoB levels and the ApoB/ApoA1 ratio were associated with higher TyG-BMI in both men and women in multivariable analysis (men_Q4 vs. Q1_: 239.559 vs. 217.091 and women_Q4 vs. Q1_: 231.823 vs. 217.063 for ApoB; men_Q4 vs. Q1_: 238.915 vs. 216.438 and women_Q4 vs. Q1_: 232.793 vs. 217.900 for ApoB/ApoA1 ratio, all *P-*trend < 0.001). Null associations between ApoA1 and TyG-BMI, in both men and women, were observed (men_Q4 vs. Q1_: 226.896 vs. 228.959, *P-*trend = 0.144; and women_Q4 vs. Q1_: 224.045 vs. 226.776, *P-*trend = 0.135). Supplementary Table S2 demonstrates inverse associations between ApoA1 levels and AIP (men_Q4 vs. Q1_: 0.383 vs. 0.490 and women_Q4 vs. Q1_: 0.313 vs. 0.457 for ApoA1). In contrast, ApoB levels and the ApoB/ApoA1 ratio were inversely associated with AIP (men_Q4 vs. Q1_: 0.504 vs. 0.316 and women_Q4 vs. Q1_: 0.431 vs. 0.285 for ApoB; men_Q4 vs. Q1_: 0.535 vs. 0.297 and women_Q4 vs. Q1_: 0.471 vs. 0.266 for ApoB/ApoA1 ratio, all *P-*trend < 0.001). Furthermore, serum ApoA1, ApoB levels, and the ApoB/ApoA1 ratio were associated with AI, as shown in Supplementary Table S3 (all *P*-trends < 0.001). The corresponding AI values in men and women were 1.969 (vs. 2.537) and 1.856 (vs. 2.501) for ApoA1; 3.118 (vs. 1.712) and 3.064 (vs. 1.541) for ApoB; and 3.213 (vs. 1.606) and 3.068 (vs. 1.431) for the ApoB/A1 ratio in the fourth quartiles compared with the first quartiles. Similar results were also observed in subgroup analyses stratified by obesity (Supplementary Tables S4–S6).

## 4 Discussion

In this hospital-based cross-sectional study of middle-aged and elderly populations, significant inverse associations were observed between ApoA1 levels and cardiometabolic indices (TyG-BMI, AIP, and AI). In contrast, positive associations were found between ApoB levels, the ApoB/A1 ratio, and these indices (TyG-BMI, AIP, and AI). These findings suggest that ApoA1, ApoB, and the ApoB/A1 ratio may serve as dependable biomarkers for evaluating cardiovascular metabolic disorders. Maintaining high ApoA1 and low ApoB levels may be beneficial for the primary prevention of cardiovascular diseases through improvement of cardiometabolic indices (TyG-BMI, AIP, and AI).

ApoA1 plays an important functional role in anti-atherosclerosis processes (10). As novel cardiometabolic indices, there is growing interest in the roles of TyG-BMI, AIP, and AI in CAD. However, data examining the associations of ApoA1 with TyG-BMI, AIP, and AI remain scarce. Notably, no prior study has directly evaluated the relationship between ApoA1 and TyG-BMI. Consistent with our results, previous studies reported that ApoA1 modulates TG levels in both children (32) and adults (25), and low ApoA1 levels were associated with insulin resistance (33) and elevated glucose levels (34). The mechanisms for the associations between ApoA1 and TyG-BMI might be explained by glucose and traditional lipid. Primarily, ApoA1 has shown to regulate glucose concentration by increasing insulin sensitivity, improving pancreatic β-cell function, and increasing insulin production and secretion (35). Animal experiments have verified that ApoA1 exerts acute improvements in insulin secretion in insulin-resistant mice (36). ApoA1 may also modulate TG concentration by influencing the lipase-mediated breakdown of TG particles (23, 37). In addition, to date, only one observational study has reported that ApoA1 plays an important role in the regulation of adverse AIP values (23). Similarly, our study also observed the inverse associations of ApoA1 with AIP and AI. Although the biological mechanism of the associations of ApoA1 with AIP and AI is uncertain, the role of ApoA1 in traditional lipids may explain these relationships. First, *in vitro and in vivo* studies indicate that ApoA1 could improve glucose tolerance and regulate insulin levels by promoting the expression of peroxisome proliferator-activated receptor *α* (38). ApoA1 may reduce adiposity and increase energy expenditure by activating PI3K-Akt-mTORC1 signaling, which negatively modulates autophagy (39). Second, ApoA1 can downregulate the expression contents of the vital adipogenic-related cytokines and decrease the intracellular TG concentration (40). Third, ApoA1 may influence TG metabolism and glucose tolerance through adenosine 5′-monophosphate-activated protein kinase-dependent mitochondrial biogenesis (41) and adenosine monophosphate-activated protein kinase complex (42). Fourth, ApoA1 can diminish lipid-binding capability (43) and facilitate insulin sensitivity in adipose tissue and skeletal muscle (44).

In addition, prior studies have demonstrated that ApoB provides important predictive value for cardiovascular risk than LDL-C (45, 46), particularly in individuals with endocrine metabolic abnormalities (e.g., diabetes mellitus, insulin resistance, obesity, and metabolic syndrome) (46). Nonetheless, no previous study had investigated the associations between ApoB and cardiometabolic indices (TyG-BMI, AIP, and AI). Our study found that ApoB was positively associated with TyG-BMI, AIP, and AI. The effect sizes were more pronounced in obese individuals than in non-obese individuals. Significant associations were more likely to be detected in obese individuals with dyslipidemia and hyperglycemia. Consistent with this, previous epidemiological data demonstrated that elevated ApoB was significantly associated with increased glucose levels (33, 47), TG (48, 49), homeostasis model assessment (50–52), obesity (53), and HDL-C levels (54). The mechanisms underlying the associations between ApoB and cardiometabolic indices may be explained by glycolipid endocrine metabolism. Dysregulation of ApoB metabolism can induce insulin resistance by suppressing the insulin signaling cascade and activating c-Jun N-terminal kinase (55, 56). Another study reported that ApoB may be related to the development of glycemic dysregulation by increasing insulin resistance and pancreatic β-cell function (57). Moreover, ApoB may restrain lipolysis in adipocytes through metabolic pathways from the liver to peripheral fat (58) and through the synthesis and secretion of very-low-density lipoprotein (59). Furthermore, previous studies have also emphasized that the ApoB/A1 ratio is a better prognosticator of cardiovascular risk compared with traditional lipid measurements (60, 61). A recent review also reported a significant relationship between elevated ApoB/A1 ratio and metabolic syndrome in children and adolescents (62). Consistent with these findings, our study demonstrated that the ApoB/A1 ratio was positively associated with cardiometabolic indices (TyG-BMI, AIP, and AI).

In addition, linear directional trends were initially found between ApoA1 and ApoB with TyG-BMI. However, effect sizes weakened when exposure reached the Q3 range, indicating that non-linear patterns may exist. Lipotoxicity and insulin resistance may be partially mitigated via endogenous estrogen-mediated lipid homeostatic mechanisms in women with moderate ApoB levels (63). Moreover, significant associations of ApoA1 and ApoB with TyG-BMI were identified in the total population, but these associations were non-significant after stratification by sex. Insufficient statistical power induced by the decreased sample size may attenuate these associations after stratification by sex. Differences in sex hormones, lifestyle, and body fat distribution between men and women may also reduce these associations by altering intra-subgroup variation of metabolic parameters.

## 5 Strengths and limitations

This study has several strengths. First, it is the first investigation to evaluate the associations of ApoA1, ApoB, and their ratio with novel cardiometabolic indices (TyG-BMI, AIP, and AI) in adults with CAD. Our findings indicated that apolipoproteins may influence the pathological process of cardiovascular disease by modulating the metabolism of glucose and lipids. Second, the relatively large population permitted us to analyze these associations efficiently. Third, consistent results for different novel cardiometabolic indices (e.g., TyG-BMI, AIP, and AI) diminished the probability of falsely significant results and ensured the reliability and robustness of the findings. Fourth, it is straightforward to calculate TyG-BMI, AIP, and AI from glucose, BMI, TG, and HDL-C since the standard assay for glucose, BMI, TG, and HDL-C were widely used in clinical practice, which could reflect comprehensively the metabolism of circulating glucose and lipid. Therefore, utilizing of ApoA1 and ApoB to regulate cardiometabolic indices (e.g., TyG-BMI, AIP, and AI) is feasible for the improvement of cardiovascular disease and its complications. Nevertheless, some limitations of this study need to be acknowledged. First, the cross-sectional design of the study limited its ability to provide causal relationships between serum ApoA1, ApoB, and the novel cardiometabolic indices. A review indicated that circulating apolipoproteins and lipids exhibited bidirectional regulatory interactions (64). Previous studies have pointed out that abnormal ApoA1 and ApoB concentrations might serve as potential indicators for metabolic syndrome (65), and metabolic syndrome were accompanied by changes in the full spectrum of apolipoproteins (e.g., ApoA1 and ApoB) (66). Meanwhile, metabolic dyslipidemia in insulin-resistant states may drive pathological modulation of apolipoproteins levels (67, 68). Thus, further research is needed to identify the underlying mechanisms of these associations. Second, CAD severity metrics and baseline medications such as statins were not taken into account in this study, which may potentially impact the results. The associations of ApoA1, ApoB, and their ratio with novel cardiometabolic indices may have been overestimated. However, the probability of false-positive results was attenuated because we included potential confounders to exclude interference in the results, including the use of lipid-lowering, hypoglycemic, and antihypotensive agents. Third, the findings of the present study were only generalizable to hospitalized Chinese individuals with CAD. The extrapolation of results may be limited because we only included patients with CAD. Thus, our findings need to be validated in community cohorts, non-CAD populations, and other ethnic groups via prospective longitudinal studies. Further prospective studies with different populations are warranted to confirm our findings. Finally, as with all observational studies, the possibility of residual confounding could not be excluded.

## 6 Conclusion

In conclusion, ApoA1 was inversely associated with novel cardiometabolic indices (e.g., TyG-BMI, AIP, and AI) in patients with CAD, while significant positive associations were observed between ApoB, the ApoB/A1 ratio, and these novel indices. Replication of these results is warranted across diverse populations in larger longitudinal studies.

## Data Availability

The original contributions presented in the study are included in the article/Supplementary Material; further inquiries can be directed to the corresponding author.
