# Supplementary material for: Associations of apolipoprotein A1, apolipoprotein B, and their ratio with novel cardiometabolic indices in adults with coronary artery disease
Source: Front Cardiovasc Med. 2026 Sep 21;13:1893486. doi: 10.3389/fcvm.2026.1893486 (PMC13635120; doi:10.3389/fcvm.2026.1893486)
Supplement: Supplementary file 1 [file Table1.doc]

**Supplemental Table 1.** The multivariable associations of ApoA1 and ApoB with TyG-BMI in both men and women (Mean ± SE).

| Variables | Quartiles by ApoA1, ApoB and its ratio | | | | *P*-trend |
| --- | --- | --- | --- | --- | --- |
|  | Q1 | Q2 | Q3 | Q4 |  |
| Male |  |  |  |  |  |
| ApoA1 | 228.959 ± 0.88 | 227.912 ± 0.91 | 228.255 ± 0.87 | 226.896 ± 0.90 | 0.144 |
|  | 228.959 ± 0.88 | 227.912 ± 0.91 | 228.255 ± 0.87 | 226.896 ± 0.90 | 0.144 |
| ApoB | 217.091 ± 0.88 | **224.482 ± 0.85^**^** | **230.839 ± 0.87^**##^** | **239.559 ± 0.87^**##¶¶^** | **<0.001** |
|  | 217.091 ± 0.88 | **224.481 ± 0.85^**^** | **230.839 ± 0.87^**##^** | **239.559 ± 0.87^**##¶¶^** | **<0.001** |
| ApoB/ApoA1 ratio | 216.440 ± 0.87 | **225.157 ± 0.87^**^** | **231.554 ± 0.87^**##^** | **238.912 ± 0.87^**##¶¶^** | **<0.001** |
|  | 216.438 ± 0.87 | **225.158 ± 0.87^**^** | **231.553 ± 0.87^**##^** | **238.915 ± 0.87^**##¶¶^** | **<0.001** |
| Female |  |  |  |  |  |
| ApoA1 | 226.790 ± 1.21 | 228.343 ± 1.20 | 228.467 ± 1.18 | 224.044 ± 1.21 | 0.133 |
|  | 226.776 ± 1.21 | 228.355 ± 1.20 | 228.467 ± 1.183 | 224.045 ± 1.211 | 0.135 |
| ApoB | 217.037 ± 1.18 | **229.006 ± 1.22^**^** | **230.002 ± 1.18^**^** | **231.831 ± 1.17^**^** | **<0.001** |
|  | 217.063 ± 1.18 | **228.977 ± 1.22^**^** | **230.010 ± 1.18^**^** | **231.823 ± 1.17^**^** | **<0.001** |
| ApoB/ApoA1 ratio | 217.880 ± 1.18 | **226.407 ± 1.19^**^** | **230.714 ± 1.19^**^** | **232.797 ± 1.19^**##^** | **<0.001** |
|  | 217.900 ± 1.18 | **226.401 ± 1.19^**^** | **230.704 ± 1.19^**^** | **232.793 ± 1.19^**##^** | **<0.001** |

Abbreviations were shown in Table 1 plus Q, quartile and SE: standard error. Age, smoking status, alcohol consumption, hypotensive, lipid-lowering and hypoglycemic agent use were adjusted.

^**^ *P* < 0.001 compared with Q1;

**^##^** *P* < 0.001 compared with Q2;

**^¶¶^** *P* < 0.001 compared with Q3.

**Supplemental Table 2.** The multivariable associations of ApoA1 and ApoB with AIP in men and women (Mean ± SE).

| Variables | Quartiles by ApoA1, ApoB and its ratio | | | | *P*-trend |
| --- | --- | --- | --- | --- | --- |
|  | Q1 | Q2 | Q3 | Q4 |  |
| Male |  |  |  |  |  |
| ApoA1 | 0.490 ± 0.01 | **0.423 ± 0.01^**^** | **0.411 ± 0.01^**^** | **0.383 ± 0.01^**##¶^** | **<0.001** |
|  | 0.490 ± 0.01 | **0.423 ± 0.01^**^** | **0.411 ± 0.01^**^** | **0.383 ± 0.01^**##¶^** | **<0.001** |
| ApoB | 0.316 ± 0.01 | **0.421 ± 0.01^**^** | **0.469 ± 0.01^**##^** | **0.504 ± 0.01^**##¶¶^** | **<0.001** |
|  | 0.316 ± 0.01 | **0.421 ± 0.01^**^** | **0.469 ± 0.01^**##^** | **0.504 ± 0.01^**##¶¶^** | **<0.001** |
| ApoB/ApoA1 ratio | 0.297 ± 0.01 | **0.405 ± 0.01^**^** | **0.471 ± 0.01^**#^** | **0.535 ± 0.01^**##¶¶^** | **<0.001** |
|  | 0.297 ± 0.01 | **0.405 ± 0.01^**^** | **0.472 ± 0.01^**##^** | **0.535 ± 0.01^**##¶¶^** | **<0.001** |
| Female |  |  |  |  |  |
| ApoA1 | 0.457 ± 0.01 | **0.405 ± 0.01^**^** | **0.360 ± 0.01^**#^** | **0.383 ± 0.01^**##¶¶^** | **<0.001** |
|  | 0.457 ± 0.01 | **0.405 ± 0.01^**^** | **0.360 ± 0.01^**#^** | **0.313 ± 0.01^**##¶¶^** | **<0.001** |
| ApoB | 0.285 ± 0.01 | **0.406 ± 0.01^**^** | **0.416 ± 0.01^**^** | **0.431 ± 0.01^**^** | **<0.001** |
|  | 0.285 ± 0.01 | **0.405 ± 0.01^**^** | **0.416 ± 0.01^**^** | **0.431 ± 0.01^**^** | **<0.001** |
| ApoB/ApoA1 ratio | 0.266 ± 0.01 | **0.378 ± 0.01^**^** | **0.421 ± 0.01^**##^** | **0.471 ± 0.01^**##¶¶^** | **<0.001** |
|  | 0.266 ± 0.01 | **0.378 ± 0.01^**^** | **0.421 ± 0.01^**#^** | **0.471 ± 0.01^**##¶¶^** | **<0.001** |

Abbreviations were shown in Table 1 plus Q, quartile and SE: standard error. Age, smoking status, alcohol consumption, hypotensive, lipid-lowering and hypoglycemic agent use were adjusted.

^**^ *P* < 0.001 compared with Q1;

**^#^** *P* < 0.05 compared with Q2;

**^##^** *P* < 0.05 compared with Q2;

**^¶^** *P* < 0.05 compared with Q3;

**^¶¶^** *P* < 0.001 compared with Q3.

**Supplemental Table 3.** The multivariable associations of ApoA1 and ApoB with AI in both men and women (Mean ± SE).

| Variables | Quartiles by ApoA1, ApoB and its ratio | | | | *P*-trend |
| --- | --- | --- | --- | --- | --- |
|  | Q1 | Q2 | Q3 | Q4 |  |
| Male |  |  |  |  |  |
| ApoA1 | 2.537 ± 0.05 | 2.495 ± 0.05 | **2.189 ± 0.05^**##^** | **1.969 ± 0.05^**##¶^** | **<0.001** |
|  | 2.537 ± 0.05 | 2.495 ± 0.05 | **2.189 ± 0.05^**##^** | **1.969 ± 0.05^**##¶^** | **<0.001** |
| ApoB | 1.712 ± 0.04 | **1.966 ± 0.04^*^** | **2.425 ± 0.04^**##^** | **3.118 ± 0.04^**##¶¶^** | **<0.001** |
|  | 1.712 ± 0.04 | **1.966 ± 0.04^*^** | **2.425 ± 0.04^**##^** | **3.118 ± 0.04^**##¶¶^** | **<0.001** |
| ApoB/ApoA1 ratio | 1.606 ± 0.04 | **2.018 ± 0.04^**^** | **2.370 ± 0.04^**##^** | **3.213 ± 0.04^**##¶¶^** | **<0.001** |
|  | 1.606 ± 0.04 | **2.018 ± 0.04^**^** | **2.370 ± 0.04^**##^** | **3.213 ± 0.04^**##¶¶^** | **<0.001** |
| Female |  |  |  |  |  |
| ApoA1 | 2.501 ± 0.06 | **2.178 ± 0.06^**^** | **2.025 ± 0.06^**^** | **1.856 ± 0.05^**##^** | **<0.001** |
|  | 2.501 ± 0.06 | **2.178 ± 0.06^**^** | **2.025 ± 0.06^**^** | **1.856 ± 0.05^**##^** | **<0.001** |
| ApoB | 1.543 ± 0.05 | **1.813 ± 0.05^**^** | **2.159 ± 0.06^**##^** | **3.064 ± 0.05^**##¶¶^** | **<0.001** |
|  | 1.541 ± 0.05 | **1.814 ± 0.05^*^** | **2.159 ± 0.06^**##^** | **3.064 ± 0.05^**##¶¶^** | **<0.001** |
| ApoB/ApoA1 ratio | 1.431 ± 0.05 | **1.836 ± 0.05^**^** | **2.231 ± 0.05^**##^** | **3.067 ± 0.054^**##¶¶^** | **<0.001** |
|  | 1.431 ± 0.05 | **1.837 ± 0.05^**^** | **2.230 ± 0.05^**##^** | **3.068 ± 0.05^**##¶¶^** | **<0.001** |

Abbreviations were shown in Table 1 plus Q, quartile and SE: standard error. Age, smoking status, alcohol consumption, hypotensive, lipid-lowering and hypoglycemic agent use were adjusted.

^*^ P < 0.05 compared with Q1;

P < 0.001 compared with Q1;

**^##^** P < 0.05 compared with Q2;

**^¶^** P < 0.05 compared with Q3;

**^¶¶^** P < 0.001 compared with Q3.

**Supplemental 4.** The multivariable associations of ApoA1 and ApoB with TyG-BMI by obesity status (Mean ± SE).

| Variables | Quartiles by ApoA1, ApoB and its ratio | | | | *P*-trend |
| --- | --- | --- | --- | --- | --- |
|  | Q1 | Q2 | Q3 | Q4 |  |
| BMI < 28 kg/m² |  |  |  |  |  |
| ApoA1 | 216.681 ± 0.63 | 216.962 ± 0.62 | 217.494 ± 0.63 | 215.909 ± 0.628 | 0.527 |
| ApoB | 207.425 ± 0.61 | **215.449 ± 0.61^**^** | **219.642 ± 0.61^**##^** | **224.574 ± 0.62^**##¶¶^** | **<0.001** |
| ApoB/ApoA1 ratio | 207.754 ± 0.61 | **214.868 ± 0.61^**^** | **220.486 ± 0.61^**##^** | **223.926 ± 0.61^**##¶¶^** | **<0.001** |
| BMI ≥ 28 kg/m² |  |  |  |  |  |
| ApoA1 | 278.234 ± 1.40 | 276.321 ± 1.40 | 278.396 ± 1.38 | **282.659 ± 1.39^#^** | **0.014** |
| ApoB | 268.911 ± 1.36 | **277.963 ± 1.37^**^** | **281.773 ± 1.35^**^** | **287.117 ± 1.37^**##^** | **<0.001** |
| ApoB/ApoA1 ratio | 270.492 ± 1.38 | **279.540 ± 1.37^**^** | **279.677 ± 1.37^**^** | **285.910 ± 1.37^**#¶^** | **<0.001** |

Abbreviations were shown in Table 1 plus Q, quartile and SE: standard error. Age, smoking status, alcohol consumption, hypotensive, lipid-lowering and hypoglycemic agents use were adjusted.

^**^ *P* < 0.001 compared with Q1;

**^#^** *P* < 0.05 compared with Q1;

**^##^** *P* < 0.001 compared with Q2;

**^¶^** *P* < 0.001 compared with Q1;

**^¶¶^** *P* < 0.001 compared with Q3.

**Supplemental 5.** The multivariable associations of ApoA1 and ApoB with AIP by obesity status (Mean ± SE).

| Variables | Quartiles by ApoA1, ApoB and its ratio | | | | *P*-trend |
| --- | --- | --- | --- | --- | --- |
|  | Q1 | Q2 | Q3 | Q4 |  |
| BMI < 28 kg/m² |  |  |  |  |  |
| ApoA1 | 0.471 ± 0.01 | 0.405 ± 0.01 | 0.383 ± 0.01 | 0.345 ± 0.01 | **<0.001** |
| ApoB | 0.293 ± 0.01 | **0.403 ± 0.01^**^** | **0.442 ± 0.01^**^** | **0.466 ± 0.01^**##¶^** | **<0.001** |
| ApoB/ApoA1 ratio | 0.276 ± 0.01 | **0.382 ± 0.01^**^** | **0.445 ± 0.01^**##^** | **0.501 ± 0.01^**##¶¶^** | **<0.001** |
| BMI ≥ 28 kg/m² |  |  |  |  |  |
| ApoA1 | 0.511 ± 0.01 | 0.473 ± 0.01 | **0.432 ± 0.01^**^** | **0.419 ± 0.01^**#^** | **<0.001** |
| ApoB | 0.353 ± 0.01 | **0.462 ± 0.01^**^** | **0.491 ± 0.01^**^** | **0.527 ± 0.01^**##^** | **<0.001** |
| ApoB/ApoA1 ratio | 0.338 ± 0.01 | **0.461 ± 0.01^**^** | **0.495 ± 0.01^**^** | **0.540 ± 0.01^**##¶^** | **<0.001** |

Abbreviations were shown in Table 1 plus Q, quartile and SE: standard error. Age, smoking status, alcohol consumption, hypotensive, lipid-lowering and hypoglycemic agent use were adjusted.

^**^ *P* < 0.001 compared with Q1;

**^#^** *P* < 0.05 compared with Q1;

**^##^** *P* < 0.001 compared with Q2;

**^¶^** *P* < 0.001 compared with Q1;

**^¶¶^** *P* < 0.001 compared with Q3.

**Supplemental 6.** The multivariable associations of ApoA1 and ApoB with AI by obesity status (Mean ± SE).

| Variables | Quartiles by ApoA1, ApoB and its ratio | | | | *P*-trend |
| --- | --- | --- | --- | --- | --- |
|  | Q1 | Q2 | Q3 | Q4 |  |
| BMI < 28 kg/m² |  |  |  |  |  |
| ApoA1 | 2.459 ± 0.04 | **2.314 ± 0.04^*^** | **2.067 ± 0.04^**##^** | **1.904 ± 0.04^**##¶^** | **<0.001** |
| ApoB | 1.588 ± 0.04 | **1.851 ± 0.04^**^** | **2.257 ± 0.04^**##^** | **3.047 ± 0.04^**##¶¶^** | **<0.001** |
| ApoB/ApoA1 ratio | 1.492 ± 0.04 | **1.904 ± 0.04^**^** | **2.246 ± 0.04^**##^** | **3.104 ± 0.04^**##¶¶^** | **<0.001** |
| BMI ≥ 28 kg/m² |  |  |  |  |  |
| ApoA1 | 2.827 ± 0.10 | 2.629 ± 0.10 | **2.463 ± 0.10^*^** | **2.022 ± 0.10^**##¶^** | **<0.001** |
| ApoB | 1.960 ± 0.10 | **2.141 ± 0.10** | **2.574 ± 0.10^**#^** | **3.270 ± 0.10^**##¶¶^** | **<0.001** |
| ApoB/ApoA1 ratio | 1.748 ± 0.10 | **2.223 ± 0.10^*^** | **2.617 ± 0.10^**#^** | **3.354 ± 0.10^**##¶¶^** | **<0.001** |

Abbreviations were shown in Table 1 plus Q, quartile and SE: standard error. Age, smoking status, alcohol consumption, hypotensive, lipid-lowering and hypoglycemic agent use were adjusted.

^**^ *P* < 0.001 compared with Q1;

**^##^** *P* < 0.001 compared with Q2;

**^¶¶^** *P* < 0.001 compared with Q3.
